# Sustainable Plastics: Effect of Bio-Based Plasticizer on Crystallization Kinetics of PLA

**DOI:** 10.3390/polym17212935

**Published:** 2025-11-01

**Authors:** David Alberto D’Amico, Liliana Beatriz Manfredi, Norma Esther Marcovich, Mirna Alejandra Mosiewicki, Viviana Paola Cyras

**Affiliations:** 1 Instituto de Investigaciones en Ciencia y Tecnología de Materiales (INTEMA), Universidad Nacional de Mar del Plata—Consejo Nacional de Investigaciones Científicas y Técnicas (CONICET), Mar del Plata C/7600, Argentina; dadamico@fi.mdp.edu.ar (D.A.D.); lbmanfre@fi.mdp.edu.ar (L.B.M.); marcovic@fi.mdp.edu.ar (N.E.M.); mirna@fi.mdp.edu.ar (M.A.M.); 2 Departamento de Ingeniería Química y en Alimentos, Facultad de Ingeniería, Universidad Nacional de Mar del Plata, Mar del Plata C/7600, Argentina

**Keywords:** bio-based plasticizer, polylactic acid (PLA), crystallization behavior

## Abstract

This work investigates the effect of a bio-based plasticizer derived from used sunflower oil on the crystallization behavior of poly (lactic acid) (PLA), comparing it with that of the conventional plasticizer tributyrin. This study aims to explore biodegradable alternatives to petroleum-based materials and to evaluate their potential in modulating PLA crystallization kinetics without altering the crystalline structure of the resulting sustainable material solutions with tailored performance. PLA-based films containing 5%, 10%, and 15% plasticizer were prepared and characterized by differential scanning calorimetry (DSC), polarized optical microscopy (POM), and X-Ray diffraction (XRD). DSC analysis showed a decrease in the glass transition temperatures upon plasticization, with tributyrin producing a more pronounced effect than the recycled sunflower oil plasticizer. XRD patterns confirmed that the crystalline form of PLA remained unchanged regardless of plasticizer type or content. POM revealed that both plasticizers influenced crystallization kinetics, with the bio-plasticizer promoting larger and more sparsely distributed spherulites than tributyrin, indicating differences in nucleation efficiency and crystal growth. Crystallization kinetics were further analyzed using the Avrami model, the Lauritzen-Hoffman theory, and the isoconversional method. Avrami analysis indicated that nucleation mechanisms were largely unaffected, although the overall crystallization rate increased upon plasticization. Lauritzen-Hoffman analysis confirmed crystallization in Regime III, controlled by nucleation, while isoconversional analysis showed reduced activation energy in plasticized PLA. These findings highlight the ability of bio-derived plasticizers to modulate PLA crystallization, promoting the valorization of a food industry residue as a sustainable plasticizer. This study hopes to contribute relevant knowledge to emerging areas of polymer processing, such as 3D printing, to develop sustainable and high-performance PLA-based materials.

## 1. Introduction

The global shift towards the development and utilization of environmentally sustainable polymers has led to a significant increase in academic research over the past three decades [1,2]. These studies have provided a foundation for the identification and comprehensive characterization of potential polymers intended to replace petroleum-derived counterparts [3,4]. However, the industrial implementation of such sustainable materials is often hampered by their suboptimal mechanical, thermal, and processing properties [5]. Consequently, the incorporation of additives to enhance these properties is imperative. To ensure the overall sustainability of the final material, these additives must also adhere to environmentally friendly principles.

Poly (lactic acid) (PLA) is a biodegradable polyester that can be synthesized from agricultural waste [6]. Its favorable properties make it a promising alternative to conventional petroleum-based polymers. Moreover, PLA is among the most extensively used polymers in additive manufacturing technologies [7], further underscoring the necessity of innovative research aimed at improving its performance.

In additive manufacturing, the formation of the final material is governed by multiple processing parameters, including bed temperature, processing time, and injection temperature [8,9]. These parameters are intrinsically linked to the final properties of the fabricated part. Furthermore, processing parameters exert a substantial influence on the crystalline architecture of the final product, which, in turn, plays a critical role in determining its physical characteristics. For instance, the transparency of a film depends on the type and dimensions of the crystalline domains formed during processing. Similarly, the mechanical behavior of the polymer, whether brittle or ductile, can be dictated by the relative prevalence of crystalline versus amorphous phases [4,10,11,12,13].

Given these considerations, a comprehensive understanding of the impact of additive incorporation on the crystalline structure and crystallization kinetics of polymeric materials is essential for optimizing their final properties. Additionally, elucidating the crystallization kinetics provides valuable insights for refining the numerous processing parameters that govern the fabrication of polymer-based components [14,15,16,17].

The main objective of this study is to explore the effect of incorporating a sustainable plasticizer derived from used sunflower oil (USOP) on the crystallization behavior of poly (lactic acid) (PLA), with a particular focus on its application in emerging polymer processing such as 3D printing. In line with the growing demand for biodegradable alternatives to petroleum-based materials, this work investigates whether bio-based plasticizers can effectively modulate PLA crystallization kinetics and morphology without altering its crystalline structure. The performance of USOP is compared with that of the commercially available biodegradable plasticizer tributyrin (TB) to evaluate how their chemical nature influences nucleation dynamics, spherulitic development, crystallization rate, and activation energy. Through a comprehensive thermal and structural characterization, this study aims to contribute to the design of functional and sustainable PLA formulations compatible with advanced manufacturing technologies and circular economy concepts.

The crystallization behavior of poly (lactic acid) (PLA) is strongly affected by the presence of plasticizers, which modify chain dynamics and, therefore, the kinetics of nucleation and crystal growth. Plasticizers reduce intermolecular interactions, increasing segmental mobility and facilitating the rearrangement of chains into ordered regions. Consequently, the addition of low-molecular-weight plasticizers tends to reduce both the glass transition (Tg) and cold-crystallization temperatures (Tcc) while improving the overall crystallization rate and degree of crystallinity under non-isothermal conditions, as well as the biodegradation rate [18,19]. These effects are often reflected in a decrease in the half-crystallization time (t_1_/_2_) and an increase in the Avrami rate constant (k), evidencing faster nucleation and crystal growth kinetics [20,21]. In addition, limited research has been conducted on the use of waste-derived plasticizers obtained from renewable sources and post-consumer waste, such as used cooking oils, to modify the PLA crystallization process. The novelty of this work lies in the valorization of a common food-industry residue as a sustainable plasticizer precursor, aiming to improve the flexibility and control the crystalline organization of PLA while supporting circular bioeconomy principles. This environmentally friendly strategy provides an innovative route for enhancing the performance of biodegradable packaging materials through upcycling approaches.

## 2. Materials and Methods

### 2.1. Materials

Poly (lactic acid) (PLA) was provided by NatureWorks LLC, Plymouth, MN, USA (INGEO 3D700). Glyceryl tributyrate, commonly referred to as tributyrin (TB), was purchased from Fluka and utilized as the standard plasticizer for PLA. A biobased plasticizer, synthesized in a prior study [22] and denoted as USOP, was derived from sunflower oil used in potato frying, supplied by a local branch of Pepsico, Buenos Aires, Argentina.

### 2.2. Film Preparation

Before processing, the PLA pellets were subjected to vacuum drying for 24 h at 50 °C in a Cole Parmer oven (Cole Parmer, Vernon Hills, IL, USA). Blends were prepared in a Haake mixer, operating at 190 °C with a screw rotation speed of 50 rpm for 3 min. PLA pellets were fed into the mixer until melted, and then the plasticizer (TB or USOP) was added. The weight ratios of PLA to plasticizer used were 95/5, 90/10, and 85/15, and the resulting films were named based on their weight composition as PLA/%TB/USOP. Additionally, neat PLA without plasticizer was processed to serve as a reference material. Films of each blend were fabricated using a hydraulic press heated to 190 °C. The samples were placed between the two press plates and held at atmospheric pressure for 1 min until melted, followed by applying a pressure of 50 MPa for 3 min.

### 2.3. Methods

#### 2.3.1. Differential Scanning Calorimetry (DSC)

Thermal analysis and crystallization kinetics were conducted using a Perkin-Elmer Pyris 1 DSC (Norwalk, CT, USA). Samples weighing 5–8 mg were used, which were hermetically sealed in aluminium pans. All tests were performed in triplicate under an inert nitrogen atmosphere (20 mL/min). The glass transition, cold crystallization and melting temperatures, were determined by heating the samples from room temperature to 200 °C, at a rate of 10 °C/min. The degree of crystallinity (Xc) was calculated using Equation (1), using the data of the first DSC scan.

(1) $$\chi_{c,i}=\frac{\left(\Delta Hm,i-\Delta Hc,i\right)}{\left({\;\Delta H}^{0}m,i\cdot \phi i\right)\cdot 100\%}$$

In Equation (1), the *i* subscript indicates the polymer in the blend, *φ* is the mass fraction of the polymer in the formulation, *ΔH_m_* is the melting enthalpy, *ΔH_c_* is the enthalpy of crystallization and ΔH_m_^0^ is the melting enthalpy of 100% crystalline PLA (93 J/g) [20].

Additionally, to determine the thermodynamic melting temperature of PLA and PLA plasticized films, the following protocol was employed: heat the sample from room temperature to 195 °C at a rate of 30 °C/min and hold for 1 min. Subsequently, the sample was rapidly cooled to a desired isothermal crystallization temperature, Tc, at a rate of 80 °C/min, and held at that temperature until 20% of the exothermic crystallization peak was reached. Then, the melting temperature of the sample crystallized under these conditions was determined by conducting a new scan from room temperature to 195 °C, at a rate of 10 °C/min.

Isothermal crystallization experiments were performed by heating the sample from room temperature to 195 °C, at a rate of 30 °C/min and maintaining the final temperature for 1 min. Then, the sample was quenched to the desired isothermal crystallization temperature, T_C_, at a cooling rate of 80 °C/min. Crystallization was assumed to be complete when the exothermic trace converged to a horizontal baseline.

#### 2.3.2. X-Ray Diffraction

X-ray diffraction tests were performed on samples of PLA, PLA/TB and PLA/USOP to study the crystalline structure of the materials. To achieve a higher resolution diffractogram, the samples were previously heated at 110 °C in an oven under a nitrogen atmosphere for 2 h to ensure the complete crystallization of PLA (annealed). The diffractograms were obtained using an X’Pert pro-diffractometer (Eindhoven, The Netherlands) operating at 40 kV and 40 mA with CuKa radiation (λ = 0.154 nm) at a scanning speed of 1.5°/min.

#### 2.3.3. Polarized Optical Microscopy

The spherulitic morphology of PLA and PLA plasticized films was examined using polarized optical microscopy (POM) with a Leica DMLB microscope (Wetzlar, Germany) equipped with crossed polarizers. Thin samples, sandwiched between two glass cover slips, were placed inside a Linkam shearing device (Surrey, UK), and the temperature was gradually increased at a rate of 20 °C/min up to 200 °C. After maintaining this temperature for 2 min to ensure complete melting, the samples were cooled at 50 °C/min to the desired crystallization temperature, T_C_ [22]. The morphological features were recorded using a Leica DC 100 video camera (Wetzlar, Germany).

## 3. Theoretical Background of Crystallization Kinetics

### 3.1. Avrami Model

In theoretical approaches addressing kinetics of crystallization, one of the main challenges is to describe the nucleation process. Avrami proposed a general framework for the transformation of the amorphous phase into growing crystalline domains, based on the concept called “extended volume” [23].

The Avrami model for isothermal processes can be described by Equation (2):

(2) $$1-\alpha (t)=\exp(-Z_{t}.t^{n})$$

where α (t) is the relative crystallinity at time t, *n* is a numerical constant representing the mechanism considering the type of nucleation and the growth dimension, and *Z_t_* is the growth rate constant involving both nucleation and the growth rate parameters [24]. The values that both *Z_t_* and *n* can adopt are presented in Table 1 [23].

### 3.2. Lauritzen Hoffman Model

This model is widely utilized due to its ability to determine both the growth regime and the nucleation constant within a specified range of crystallization temperatures [25]. Equation (3) shows the exponential expression of the Lauritzen-Hoffman model:

(3) $$G=Go\cdot \exp\left(\frac{-U}{R\left(T-T_{\infty }\right)}\right)\cdot \exp\left(\frac{-K_{G}}{fT\Delta T}\right)$$

where *G* is the global crystallization rate (G = 1/t_1/2_), t_1/2_ is the time required to reach 50% of the final crystallinity, Go is a constant independent of temperature, U (4120 cal/mol) [20] is the activation energy for the transport of the crystallizable segments, T_∞_ is a hypothetical temperature at which molecular motion associated with viscous flow stops and is related to the glass transition temperature (T_g_) by T_∞_ = T_g_ − C, where C is a constant; ΔT: is the degree of subcooling given by Tm − T_C_; Tm is the equilibrium melting temperature; *f* is a factor that represents the variation in the enthalpy in the melt per unit volume with temperature, given by *f* = 2T/(Tm + T) and K_G_ is the nucleation constant.

### 3.3. Isoconversional Method

Isoconversional methods are often referred to as “model-free methods” due to their lack of assumptions about the mechanisms involved in sample crystallization. These methods provide insights into the variation in effective activation energy (Eα) as a function of the degree of conversion (α). The general guidelines for applying these methods have been established by the International Confederation for Thermal Analysis and Calorimetry (ICTAC) [26].

Any solid-state kinetic can be expressed as a single step kinetic equation:

(4) $$\frac{d\alpha }{dt}=k(T)\;f(\alpha )$$

where *t* is time, *T* is temperature, *k* is rate constant, *f*(*α*) is a differential form of the kinetic model and *α* is the degree of conversion.

When applying an isoconversional method, the reaction rate at a constant degree of conversion should depend solely on temperature. Under this condition, Equation (5) is obtained by taking the logarithmic derivative of the reaction rate (Equation (4)) at a fixed α.

(5) ∂lndαdt∂T−1α=∂lnk(T)∂T−1α+∂lnf(α)∂T−1α

In Equation (5), the subscript *α* indicates isoconversional values, i.e., the values related to a given degree of conversion. Isoconversional analysis for an isothermal process can be performed by modifying Equation (5), leading to Equation (6):

(6) $$\ln t_{\alpha ,i}=\ln\left[\frac{g(\alpha )}{A}\right]+\frac{E_{\alpha }}{RT_{i}}$$

where *g*(*α*) is the integral form of the kinetic model, *t* is the time, *T* is the temperature, A is a constant, *R* is the gas constant and *E_α_* is the isoconversional effective activation energy. From the slope of the plot of ln t_α,i_ vs. 1/*T_i_*, *E_α_* values of the samples can be obtained at each *α*, without assuming a kinetic model.

## 4. Results and Discussion

The inclusion of bio-derived and conventional plasticizers can influence not only the thermal transitions of PLA, but also its morphological organization and crystalline structure, which in turn determines the processing behavior and final performance of the material. Therefore, it is necessary to characterize the prepared PLA-based films to elucidate the relationship between plasticizer type, structural features, and crystallization behavior. Consequently, a combination of complementary analytical techniques was applied to provide a comprehensive description of the systems under study. In our previous work [22], we investigated the mechanical properties and thermal decomposition of the materials considered in the present study. The results showed that both the tensile strength and the elastic modulus decreased upon addition of plasticizer, while, as expected, the elongation at break increased several times compared to the unplasticized material. These observations are consistent with the typical behavior of plasticized PLA systems. Furthermore, the thermal degradation was not affected by the addition of plasticizers up to 10 wt%, while for materials containing 15 wt% plasticizers, the temperature corresponding to the maximum degradation rate slightly decreased, indicating a minor impact of the higher plasticizer content on the thermal decomposition of the material.

### 4.1. Thermal Behavior and Crystalline Structure of PLA and PLA Plasticized Films DSC

**Figure 1:**
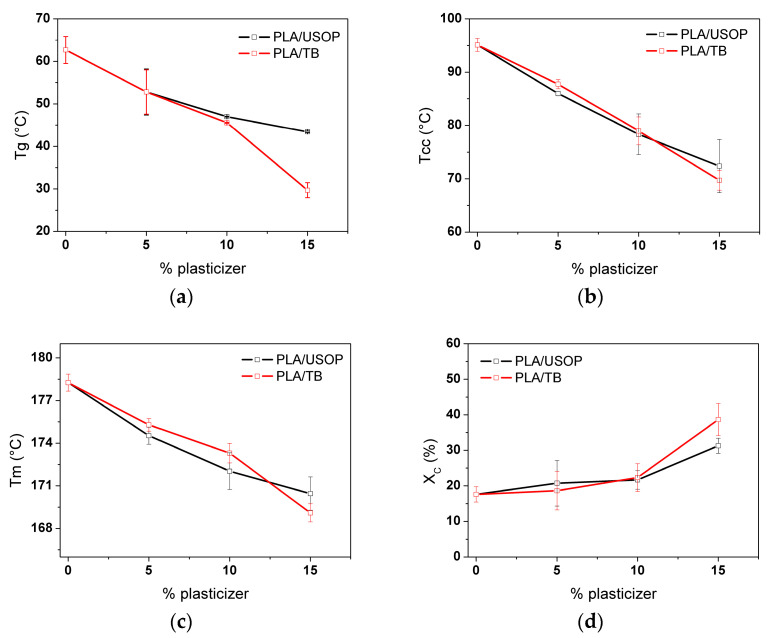
DSC results obtained from first scan of PLA and PLA plasticized materials: (**a**) Glass transition temperature (Tg), (**b**) Cold crystallization temperature (Tcc), (**c**) Melting temperature (Tm), (**d**) Crystallinity degree (X_c_).

Differential scanning calorimetry (DSC) was used to quantify the plasticizing effect of both TB and USOP, on the glass transition temperature, cold crystallization, and melting behavior, and to gather information on changes in chain mobility and crystallization kinetics. To ensure that only the effect of material processing was examined, solely the first DSC heating scan was considered. Figure 1 illustrates the variations in glass transition temperature (a), cold crystallization temperature (b), melting temperature (c) and degree of crystallinity (d), for the different formulations. The plasticization effect (Tg depression) on the matrix was evident for both USOP and TB. Although USOP and TB have similar molecular weights, their topology differ significantly. On the one hand, USOP is a linear molecule that could tend to thread into the PLA chains, hindering their nucleation; while on the other hand, TB is a drop-shaped molecule, which would act as a nucleating agent and would not have the ability to thread into the PLA chains. A similar behavior, although employing different plasticizers, was also observed by Ruiz et al. [27]. Their findings indicated that the spherulitic growth rate, nucleation rate, and overall crystallization kinetics of PLA were significantly influenced by the topology of the plasticizers chains. According to these researchers [27], the linear additive, behaved solely as a typical plasticizer for the PLA matrix, whereas the cyclic molecule showed a synergistic effect not only in accelerating PLA crystal growth but also its nucleation.

However, it should be noted that the plasticizer concentration also plays a preponderant role in determining these properties: from Figure 1 it is clear that both plasticizers behave in a similar manner at relatively low concentrations (<10% by weight), while at higher concentrations (>10 wt%), TB affects the structure and molecular orientation of the PLA chains more severely than USOP, leading to a greater reduction in molecular interactions. Other authors also reported differences in the PLA crystallization behavior with the concentration of the plasticizer [27].

Additionally, this plasticizing effect influences the cold crystallization temperature (Tcc) and melting temperature (Tm). The increased mobility of the plasticized PLA polymer chains allows crystallization to occur at lower temperatures, as evidenced by the reduction in Tcc as the plasticizer content (USOP or TB) increases. In this case, however, the threading effect attributed to the USOP can be offset by its plasticizing action that enhances diffusion. This effect results in a lower decrease in the Tcc at high plasticizing contents compared with TB, where the effect is associated with a higher nucleation density. As expected, the degree of crystallinity increases as the plasticizer content increases. However, in the case of PLA/15 TB, the lower Tcc results in a higher crystallinity degree compared to PLA/15 USOP, suggesting that TB promotes a more efficient crystallization process under these conditions.

The differences in the melting temperatures observed in the plasticized samples could indicate that crystal formation in the presence of the two plasticizers follows different kinetics mechanisms, or that the crystals formed differ in their degree of ordering. This aspect will be analyzed in more detail in the following sections.

### 4.2. X-Ray Diffraction (XRD)

**Figure 2:**
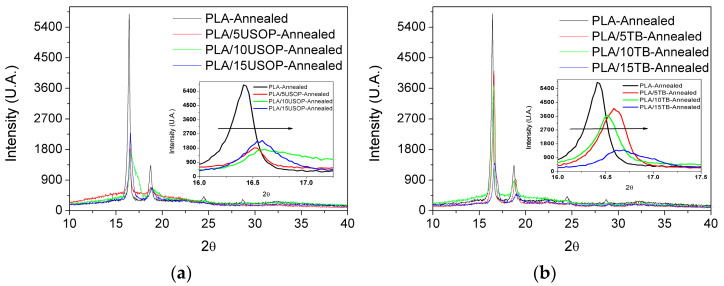
DRX patterns of: (**a**) annealed PLA and PLA/USOP plasticized materials and (**b**) annealed PLA and PLA/USOP plasticized materials. The arrow indicates the shift in 2θ.

X-ray diffraction was used to determine the crystalline form and degree of order, while confirming that the fundamental crystalline structure of PLA was preserved after plasticization. Together, DSC and X-Ray techniques provide a framework for understanding how USOP and TB influence the thermal and morphological characteristics of PLA, thereby guiding the design of sustainable materials with tailored properties. Figure 2a displays the diffractograms for the formulations containing USOP, where several diffraction peaks characteristic of PLA α’ crystalline form [28], are observed superimposed on the amorphous halo. A similar behavior was observed for the TB-plasticized materials (Figure 2b). An enlarged section of the diffraction patterns, corresponding to the (200) plane (16–18° range), is also shown in Figure 2a,b. This enlarged region reveals a shift in the main peak towards higher angles as the plasticizer content increases, indicating changes in the unit cell dimensions in which the system crystallizes. Higher 2θ values correspond to shorter interplanar spacing, which is associated with lower Tm values and is also consistent with the trend observed in the DSC section. These results confirm that none of the plasticizers alters the crystalline form of PLA; however, they do influence its crystalline structure, leading to the formation of smaller crystals.

### 4.3. Polarized Optical Microscopy (POM)

When optical microscopy with cross-polarizers is used in conjunction with a controlled heating/cooling cell, it is possible to observe the morphological characteristics of spherulites during their formation from the melt for a specified temperature program. POM enables real-time observation of spherulitic growth and size distribution, facilitating the assessment of nucleation efficiency and crystal growth dynamics.

**Figure 3:**
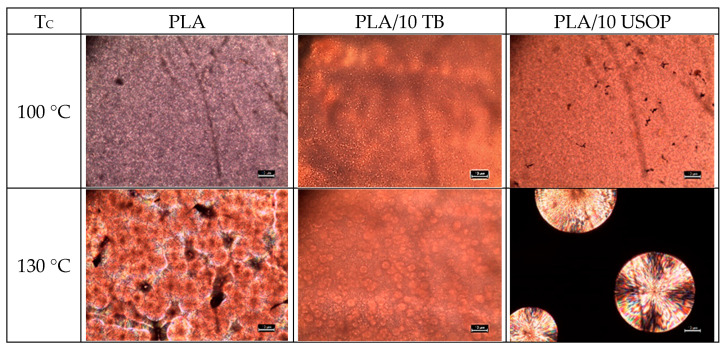
POM images after crystallization from the melt for 5 min, at different Tc for PLA, PLA/10 TB and PLA/10 USOP. The scale bar represents 10 µm.

In the case of PLA and PLA plasticized films, two crystallization temperatures (100 and 130 °C), were used to evaluate the characteristics of the spherulites formed at each T_C_. These temperatures were chosen depending on whether the objective was to promote nucleation (T_C_ = 100 °C) or spherulite growth (T_C_ = 130 °C). Furthermore, these parameters were determined by directly examining the number of spherulites formed, which reflects the nucleation capacity of the PLA chains, and by assessing the spherulite size, which is directly related to the diffusion of the PLA chains toward the core of an already formed spherulite. Using both USOP and TB as plasticizers, it is observed that the lower the crystallization temperature (i.e., the higher the undercooling), result in a higher number of spherulites, which is expected from a kinetic and thermodynamic point of view. This can be clearly observed in Figure 3, where at T_C_ = 100 °C, the spherulites exhibit an extensive overlapping and are small in diameter, making them difficult to individualize. Furthermore, there is no clear effect of the nature of the plasticizer on the crystallization of PLA at this relatively low temperature. However, at T_C_ = 130 °C, not only the spherulites can be distinguished individually in all the samples, but clear differences in their number and size can also be observed. As shown in Figure 3, the PLA/10 USOP formulation exhibits the lowest amount of spherulites, and therefore the largest crystals, followed by neat PLA with intermediate numbers and sizes, and PLA/10 TB which exhibits a higher number of relatively small spherulites. This behavior confirms both the nucleating effect credited to the star-shaped structure of TB plasticizer as well as the ability of USOP to thread into the PLA chains. The higher nucleation density observed in the PLA/TB samples compared to the neat ones, was attributed to the reduction in the nucleation energy barrier induced by the plasticizer, which enhances the mobility of the polymer chains [14,15,16,17].

### 4.4. Equilibrium Melting Temperature

To better understand into plasticizer incorporation influences the crystallite perfection of PLA, the equilibrium melting temperature (Tm^0^) was determined. This parameter represents the theoretical melting point of an infinitely large, defect-free crystal and serves as a reference point for evaluating the effect of structural imperfections introduced during crystallization. By comparing Tm^0^ values for neat and plasticized PLA, it is possible to assess whether additives modify lamellar thickness, chain-folding regularity, or nucleation behavior, thereby affecting the thermodynamic stability of the crystalline phase. Therefore, the effect of different plasticizers on the Tm^0^ of PLA was evaluated using the Hoffman and Weeks method. For this purpose, the Hoffman and Weeks equation (Equation (7)) was applied [29]:

(7) $$Tm=Tm^{0}\left(1-\frac{1}{\gamma }\right)+\frac{T_{c}}{\gamma }$$

where γ is a factor that depends on the final lamellar thickness. It is accepted that γ = l/l*, where l and l* are the thicknesses of a mature crystal and a crystal with critical diameter, respectively. Tm^0^ was determined at the point where Tm = T_C_, by plotting Tm as a function of Tc.

**Figure 4:**
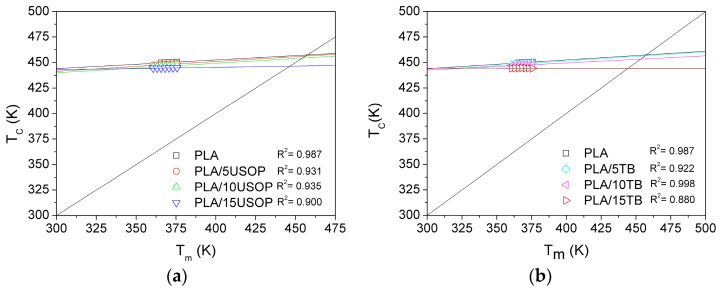
Extrapolation and fitting of the Hoffman and Weeks Equation for: (**a**) PLA and PLA/USOP plasticized materials (**b**) PLA and PLA/TB plasticized materials.

According to Figure 4, the Tm^0^ of the pristine PLA used in this work as reference sample is 184 °C. Xiao et al. [30] reported a Tm^0^ value for PLA close to 193 °C, which was intermediate compared with other values previously reported in the literature, in which the equilibrium melting temperature of PLA has been reported to range between 159.5 and 215 °C [20,31]. This disparity in the reported values could be due to the different experimental methodologies used for their determination, but also to the differences in the grade, purity and/or molecular weight among the PLAs tested.

On the other hand, the values of Tm^0^ of all plasticized formulations are lower than that of the neat PLA, denoting that the crystalline phase is less perfect than in pristine PLA. This phenomenon may be due to the development of heterogeneous nucleation induced by the addition of plasticizers, which would increase the supercooling required to form a nucleus at a fixed Tc, thereby resulting in a decrease in the regularity of the formed crystals. On the other hand, both plasticizers incorporated into PLA occupy a certain volume that could hinder the PLA chains folding process, leading to less perfect crystals, thus decreasing the thermodynamic melting temperature [32].

### 4.5. Crystallization Kinetics

To study the crystallization kinetics of PLA plasticized with USOP and TB, several kinetic models were used. All models rely on the degree of crystallinity, which can be obtained from isothermal experiments conducted at different T_C_. The relative crystallinity over time was obtained using Equation (8):

(8) $$\alpha (t)=\frac{\int_{t_{0}}^{t}\Delta H(t)}{\int_{t_{0}}^{t_{\infty }}\Delta H(t)}$$

where α(t) is the relative crystallinity of the polymer at time t, t_0_ is the time at which the crystallization process begins, t_∞_ is the time at which the crystallization process ends, and ∆H(t) is the enthalpy of crystallization.

**Figure 5:**
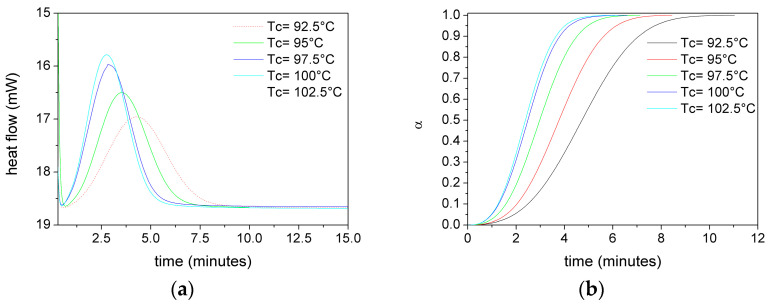
DSC heat flow of unplasticized PLA at several Tc (**a**), and (**b**) relative crystallinity of unplasticized PLA at several Tc.

Figure 5 shows, as an example, the heat flux versus time (a) and relative crystallinity (α) versus time (b) curves at different T_C_ for PLA films without plasticizer.

#### 4.5.1. Avrami Model

**Figure 6:**
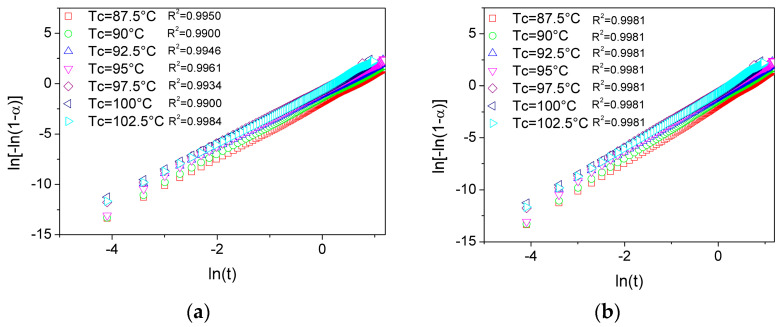
Linearization of the Avrami equation for (**a**) PLA/10 USOP and (**b**) PLA/10 TB.

To gain deeper insight into the evolution of crystallinity during isothermal crystallization, the Avrami model was applied to analyze the crystallization kinetics of PLA and its plasticized blends. As shown in Figure 6a,b, the experimental data plotted using the linearized form of Equation (2) exhibit an excellent alignment, indicating that the proposed linearization provides a valid and consistent representation of all investigated systems. The kinetic parameters obtained for each formulation, including the Avrami exponent (n) and the overall crystallization rate constant (*Z_t_*), are summarized in Table 2.

An average Avrami exponent (n) of 2.83 was obtained for neat PLA. This value corresponds to the mean of the individual *n* values calculated for each isothermal condition, which showed minimal variation. Such dispersion likely arises from the simultaneous presence of different nucleation mechanisms and crystal growth geometries during the crystallization process. In the case of pure PLA, the absence of nucleating agents would suggest a predominance of homogeneous nucleation. According to the *n* values derived from Equation (5), the crystallization process can be characterized by a combination of two- and three-dimensional spherulitic growth [33]. The values of the average Avrami exponent obtained falls within the range reported in the literature for the isothermal crystallization of PLA [34]. Pantani et al. [35] obtained similar results when studying the crystallization kinetics of virgin and processed PLA by means of extrusion and injection molding. They also reported a spherical growth of heterogeneously nucleated crystals, obtaining a good description of the kinetic processes using single values of the Avrami index equal to 2.8 and 2.7 for virgin and extruded PLA, respectively.

For the plasticized PLA systems, both with USOP and TB, the *n* values are slightly lower although comparable to those of the neat polymer. This indicates that the incorporation of any of the plasticizers does not significantly alter the fundamental crystallization mechanism of PLA. Therefore, nucleation in these systems is also expected to proceed predominantly via homogeneous pathways, with growth continuing in two and three dimensions as in the unmodified polymer.

In contrast, some differences were observed in the crystallization rate constant (Z_t_). The values for plasticized PLA films were higher than those of neat PLA, suggesting an enhanced overall crystallization process. For most materials, an increase in temperature led to higher Z_t_ values, which is consistent with the expected trend that higher temperatures promote faster crystallization. However, for the PLA/15 TB formulation, Z_t_ decreased with increasing temperature, revealing an anti-Arrhenius behavior in which the crystallization rate decreases as the temperature increases. This particular behavior will be further discussed in the following sections. On the other hand, it is important to note that such a difference was not reflected in the crystallization rates determined using the Lauritzen–Hoffman model, as will be discussed in the next section. This discrepancy highlights that Z_t_ incorporates contributions from both nucleation and growth, and therefore, it does not directly represent the global crystallization rate [33,36]. Consequently, caution should be exercised when interpreting *Z_t_* in isolation, as it may reflect complex kinetic interactions influenced by the presence of plasticizers.

#### 4.5.2. Lauritzen Hoffman Model

**Figure 7:**
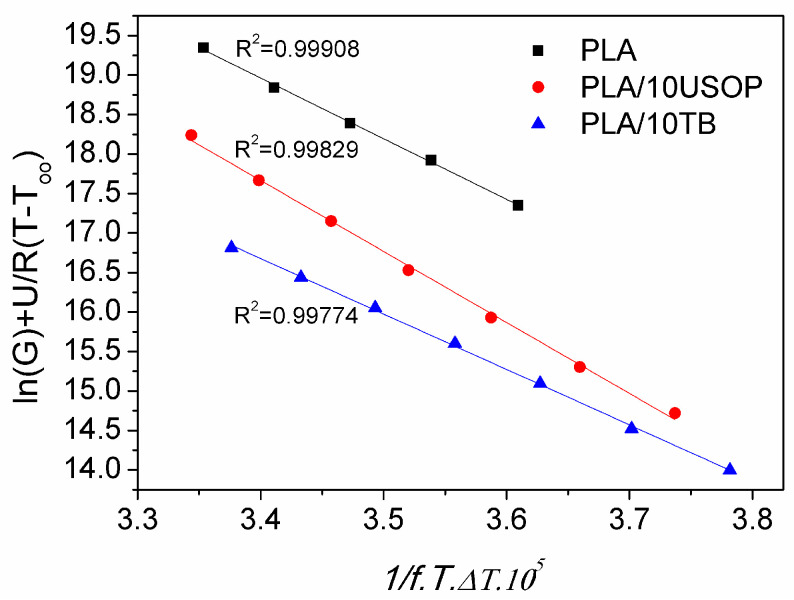
Lauritzen-Hoffman plots for PLA and PLA plasticized with 10% of TB and 10% of USOP.

To investigate and quantify the dependence of the crystallization rate on temperature, the Lauritzen-Hoffman model was applied. The model fit (Equation (3)) for neat PLA and PLA containing 10% plasticizers is presented in Figure 7. In this approach, the crystallization rate, G, is defined as the inverse of the time required to reach 50% of the final crystallinity (t_1/2_) obtained from DSC measurements (Figure 5b). The same procedure was applied to determine t_1/2_ of all plasticized systems studied. Therefore, G provides a direct measure of the overall crystallization rate, allowing comparison between neat and plasticized PLA formulations in the context of the Lauritzen-Hoffman theoretical framework [3]. The correlation of the experimental data with the model was excellent, presenting R^2^ values greater than 0.99 for the 7 data sets analyzed. In the temperature range used for the isothermal tests, no changes in the crystallization regime were observed for either PLA or any of the formulations. Comparison of the obtained values with those reported in the literature [20] indicates that the materials crystallize in Regime III, in which supercooling is such that nucleation predominates over crystal growth, since the crystals cannot grow laterally due to the high nucleation rate.

Table 3 presents the values of the nucleation constant for all the materials studied. Consistently with the results obtained by POM, PLA/10 USOP has the highest K_G_ value, indicating the lowest nucleation rate compared to the rest of the materials studied in this work and for the analyzed temperature range.

**Figure 8:**
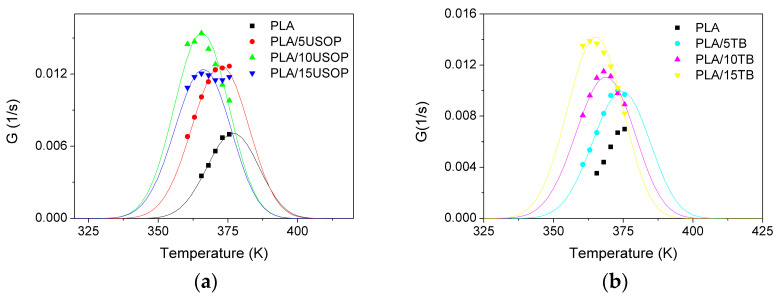
Crystallization rate vs. temperature for PLA and plasticized PLA with different percentages of: (**a**) USOP, and (**b**) TB.

Based on the calculated K_G_ values, the dependence of the overall crystallization rate as a function of temperature was plotted (Figure 8a,b) and the experimentally obtained data (1/t_1/2_) were overlapped. This type of graph is very useful for understanding the behavior of the material at different temperatures and thus outlining strategies and operating conditions for its processing.

From Figure 8, it can be observed that the materials containing both plasticizers exhibit similar behavior to that of neat PLA over the entire temperature range in which it can crystallize. In the case of materials with TB, the temperature corresponding to the maximum crystallization rate shifts to lower values compared to pure PLA, and at the same time, the overall crystallization rate increases as the TB percentage increases. This trend is consistent with the increased mobility of the plasticized polymer chains and their higher nucleation rate. In contrast, for the USOP-plasticized materials, a less pronounced effect on the temperature of the maximum rate is observed, which also agrees with the lower plasticizing effect provided by this additive and previously reported by the group [22].

#### 4.5.3. Isoconversional Method

**Figure 9:**
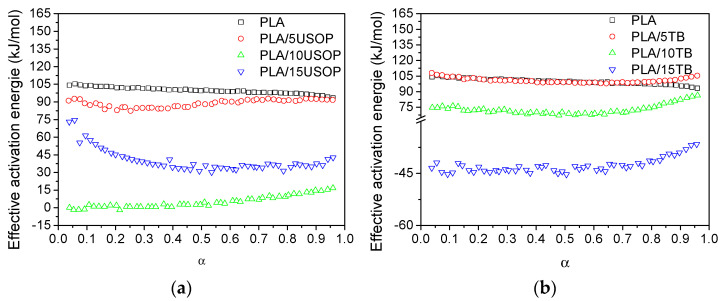
Effective activation energy profiles for: (**a**) PLA and PLA/USOP plasticized materials and (**b**) PLA and PLA/TB plasticized materials.

Figure 9a,b displays the activation energy (*Ea*) profiles for PLA, PLA/USOP, and PLA/TB samples. Throughout the crystallization process, no significant changes in the mechanism were observed, as the activation energy remained relatively stable for all materials. For the USOP-containing materials, the activation energy was consistently lower than that of the pure PLA, indicating that, within the analyzed temperature range, these materials reach their maximum crystallization rate more rapidly than PLA. A similar trend was also observed for PLA plasticized with TB.

For materials containing 10% USOP and 15% TB, negative activation energy values were observed, which are indicative of anti-Arrhenius behavior [37]. This phenomenon suggests that, within the analyzed temperature range, an increase in temperature results in a decrease in the crystallization rate. Such behavior is atypical compared to conventional crystallization processes, where an increase in temperature usually accelerates crystallization. This alignment with an anti-Arrhenius trend implies that the crystallization process is thermodynamically hindered at higher temperatures. This effect could be attributed to specific interactions between the plasticizers (USOP and TB) and the polymeric matrix, which may alter the typical crystallization mechanism, resulting in modifications to nucleation and/or crystal growth processes, which take place when additive concentrations exceed a threshold. Understanding these effects is crucial for tailoring the crystallization kinetics of PLA-based materials for specific applications, especially in 3D printing, where precise control over crystallization is essential for achieving the desired mechanical and thermal properties.

## 5. Conclusions

This study demonstrates that both the bio-based plasticizer derived from waste sunflower oil (USOP) and the conventional plasticizer tributyrin (TB) effectively enhance the crystallization rate of PLA without altering its crystalline structure. DSC, POM, and XRD analyses confirmed that plasticization lowers the glass transition temperature and influences spherulitic morphology, with USOP promoting larger and more sparsely distributed spherulites compared to TB, indicating differences in nucleation efficiency and crystal growth.

Kinetic evaluations using the Avrami model, Lauritzen–Hoffman theory, and isoconversional analysis revealed that plasticization accelerates crystallization under nucleation-controlled (Regime III) conditions while reducing the activation energy required for crystal formation. These findings confirm that USOP has comparable performance to TB in modulating PLA crystallization, despite being derived from an industrial residue.

From a practical perspective, the use of USOP provides a dual environmental benefit: it valorizes agro-industrial waste and offers a sustainable, and biodegradable alternative to petroleum-based materials. This positions USOP as a viable additive for replacing fossil-derived plasticizers in PLA-based formulations. Future research should explore production scale-up, evaluate long-term stability, and test mechanical and barrier properties of USOP-plasticized PLA under real-use conditions to enable industrial adoption.

In summary, the results confirm that bio-based plasticizers derived from waste cooking oil effectively improve the flexibility and crystallization behavior of PLA without compromising its biodegradability. The observed enhancement in chain mobility and crystallization kinetics indicates that these additives can modulate the structural organization of PLA, enabling the tuning of its thermal and mechanical performance for specific end uses. Beyond their laboratory-scale validation, these findings highlight the potential of this approach for industrial applications, particularly for the development of biodegradable packaging materials where controlled flexibility and processability are required.

## Figures and Tables

**Table 1:** Possible values for the coefficient *n* and expressions for the constant *Z_t_*.

Growth Dimensions	Instantaneous Nucleation		Spontaneous Nucleation	
	*n*	*Z_t_*	*n*	*Z_t_*
2	2	*πDG^2^*	3	*πBG^3^/3*
3	3	*4πDG^3^/3*	4	*πBG^4^/3*

*D*, instantaneous nucleation density; *B*, spontaneous nucleation rate constant; *G*, time-dependent spherulite growth rate.

**Table 2:** Avrami model parameters.

Material	T_C_ (°C)	n_Avrami_	Z_t_ = zt^n^	t_1/2_
PLA	92.5	2.75	0.0093	4.717
	95.0	2.93	0.0138	3.783
	97.5	2.86	0.0573	2.983
	100.0	2.76	0.0561	2.483
	102.5	2.85	0.0304	2.383
PLA/5 USOP	87.5	2.61	0.0625	2.450
	90.0	2.72	0.1048	1.983
	92.5	2.60	0.1886	1.650
	95.0	2.63	0.2429	1.467
	97.5	2.76	0.3036	1.350
	100.0	2.72	0.3245	1.333
	102.5	2.53	0.3705	1.317
PLA/10 USOP	87.5	2.59	0.1444	1.150
	90.0	2.63	0.2137	1.133
	92.5	2.57	0.3377	1.083
	95.0	2.57	0.3884	1.183
	97.5	2.59	0.5343	1.300
	100.0	2.57	0.5170	1.500
	102.5	2.77	0.5345	1.700
PLA/15 USOP	87.5	2.84	0.2005	1.533
	90.0	2.70	0.2842	1.417
	92.5	2.57	0.3247	1.383
	95.0	2.42	0.3401	1.400
	97.5	2.61	0.3094	1.450
	100.0	2.54	0.3223	1.450
	102.5	2.78	0.3110	1.417
PLA/5 TB	87.5	2.74	0.0164	3.950
	90.0	2.63	0.2137	3.117
	92.5	2.84	0.0548	2.484
	95.0	2.76	0.1102	2.033
	97.5	2.73	0.1759	1.733
	100.0	2.69	0.1851	1.667
	102.5	2.50	0.1926	1.717
PLA/10 TB	87.5	2.62	0.0956	2.067
	90.0	2.63	0.1518	1.733
	92.5	2.67	0.2348	1.517
	95.0	2.76	0.1102	1.450
	97.5	2.71	0.2738	1.500
	100.0	2.61	0.2094	1.700
	102.5	2.58	0.1677	1.867
PLA/15 TB	87.5	2.63	0.4030	1.233
	90.0	2.68	0.4395	1.200
	92.5	2.72	0.4404	1.217
	95.0	2.79	0.3836	1.283
	97.5	2.87	0.3209	1.400
	100.0	2.67	0.2176	1.633
	102.5	2.71	0.1226	2.030

**Table 3:** Nucleation constant K_G_ for PLA/Plasticizer.

Sample	K_G_ 10^−5^ (K^2^)
PLA	7.68
PLA/5 USOP	7.39
PLA/10 USOP	8.99
PLA/15 USOP	6.59
PLA/5 TB	6.81
PLA/10 TB	7.02
PLA/15 TB	6.36

## Data Availability

The original contributions presented in this study are included in the article. Further inquiries can be directed to the corresponding authors.

## References

[1] Mukherjee C., Varghese D., Krishna J.S., Boominathan T., Rakeshkumar R., Dineshkumar S., Brahmananda Rao C.V.S., Sivaramakrishna A. (2023). Recent Advances in Biodegradable Polymers—Properties, Applications and Future Prospects. Eur. Polym. J..

[2] Reddy C.S.K., Ghai R., Kalia V.C. (2003). Polyhydroxyalkanoates: An Overview. Bioresour. Technol..

[3] D’Amico D.A., Manfredi L.B., Cyras V.P. (2012). Relationship between Thermal Properties, Morphology, and Crystallinity of Nanocomposites Based on Polyhydroxybutyrate. J. Appl. Polym. Sci..

[4] Zhao X., Yu J., Liang X., Huang Z., Li J., Peng S. (2023). Crystallization Behaviors Regulations and Mechanical Performances Enhancement Approaches of Polylactic Acid (PLA) Biodegradable Materials Modified by Organic Nucleating Agents. Int. J. Biol. Macromol..

[5] Naeem S., Najeeb J., Usman S.M., Rafique H., Ali G.A.M., Makhlouf A.S.H. (2022). Biodegradable Polymers Challenges. Handbook of Biodegradable Materials.

[6] Moldovan A., Cuc S., Prodan D., Rusu M., Popa D., Taut A.C., Petean I., Bomboş D., Doukeh R., Nemes O. (2023). Development and Characterization of Polylactic Acid (PLA)-Based Nanocomposites Used for Food Packaging. Polymers.

[7] Joseph T.M., Kallingal A., Suresh A.M., Mahapatra D.K., Hasanin M.S., Haponiuk J., Thomas S. (2023). 3D Printing of Polylactic Acid: Recent Advances and Opportunities. Int. J. Adv. Manuf. Technol..

[8] Ligon S.C., Liska R., Stampfl J., Gurr M., Mülhaupt R. (2017). Polymers for 3D Printing and Customized Additive Manufacturing. Chem. Rev..

[9] Nadgorny M., Ameli A. (2018). Functional Polymers and Nanocomposites for 3D Printing of Smart Structures and Devices. ACS Appl. Mater. Interfaces.

[10] Molinari G., Aliotta L., Gemmi M., Lazzeri A., Righetti M.C. (2024). Constrained Amorphous Interphase in Plasticized Poly(Lactic Acid): Composition and Tensile Elastic Modulus Estimation. Polym. Test..

[11] Litauszki K., Petrény R., Haramia Z., Mészáros L. (2023). Combined Effects of Plasticizers and D-Lactide Content on the Mechanical and Morphological Behavior of Polylactic Acid. Heliyon.

[12] Li X., Shang X., Lyu J., Tong Y., Situ W., Yu L., Wu T., Xie H., Qu J. (2023). Efficient Fabrication of PLA/PHB Composites with Enhanced Mechanical Properties, Excellent Thermal Stability, Fast Crystallization Ability, and Degradation Rate via the Synergistic of Weak Shear Field and Melt Quenching Technique. Ind. Crops Prod..

[13] Tábi T., Ageyeva T., Kovács J.G. (2022). The Influence of Nucleating Agents, Plasticizers, and Molding Conditions on the Properties of Injection Molded PLA Products. Mater. Today Commun..

[14] Brüster B., Montesinos A., Reumaux P., Pérez-Camargo R.A., Mugica A., Zubitur M., Müller A.J., Dubois P., Addiego F. (2018). Crystallization Kinetics of Polylactide: Reactive Plasticization and Reprocessing Effects. Polym. Degrad. Stab..

[15] Niu D., Shen T., Xu P., Yu M., Liu T., Yang W., Wang Z., Ma P. (2023). Enhanced Crystallization, Heat Resistance and Transparency of Poly(Lactic Acid) with Self-Assembling Bis-Amide Nucleator. Int. J. Biol. Macromol..

[16] Bajwa D., Eichers M., Shojaeiarani J., Kallmeyer A. (2021). Influence of Biobased Plasticizers on 3D Printed Polylactic Acid Composites Filled with Sustainable Biofiller. Ind. Crops Prod..

[17] Chen Q., Auras R., Corredig M., Kirkensgaard J.J.K., Mamakhel A., Uysal-Unalan I. (2022). New Opportunities for Sustainable Bioplastic Development: Tailorable Polymorphic and Three-Phase Crystallization of Stereocomplex Polylactide by Layered Double Hydroxide. Int. J. Biol. Macromol..

[18] Mastalygina E.E., Aleksanyan K.V. (2023). Recent Approaches to the Plasticization of Poly(Lactic Acid) (PLA) (A Review). Polymers.

[19] Brdlík P., Novák J., Borůvka M., Běhálek L., Lenfeld P. (2022). The Influence of Plasticizers and Accelerated Ageing on Biodegradation of PLA under Controlled Composting Conditions. Polymers.

[20] Saeidlou S., Huneault M.A., Li H., Park C.B. (2012). Poly(Lactic Acid) Crystallization. Prog. Polym. Sci..

[21] Zhao X., Hu H., Wang X., Yu X., Zhou W., Peng S. (2020). Super Tough Poly(Lactic Acid) Blends: A Comprehensive Review. RSC Adv..

[22] D’Amico D.A., Hernández E., Iglesias Montes M.L., Marcovich N.E., Manfredi L.B., Cyras V.P., Mosiewicki M.A. (2024). Repurpose of Used Frying Sunflower Oil as an Ecofriendly Plasticizer for Polylactic Acid. Ind. Crops Prod..

[23] Piorkowska E., Galeski A., Haudin J.-M. (2006). Critical Assessment of Overall Crystallization Kinetics Theories and Predictions. Prog. Polym. Sci..

[24] Xiao H., Lu W., Yeh J. (2009). Effect of Plasticizer on the Crystallization Behavior of Poly(Lactic Acid). J. Appl. Polym. Sci..

[25] D’Amico D.A., Manfredi L.B., Cyras V.P. (2012). Crystallization Behavior of Poly(3-Hydroxybutyrate) Nanocomposites Based on Modified Clays: Effect of Organic Modifiers. Thermochim. Acta.

[26] Vyazovkin S., Burnham A.K., Criado J.M., Pérez-Maqueda L.A., Popescu C., Sbirrazzuoli N. (2011). ICTAC Kinetics Committee Recommendations for Performing Kinetic Computations on Thermal Analysis Data. Thermochim. Acta.

[27] Ruiz M.B., Pérez-Camargo R.A., López J.V., Penott-Chang E., Múgica A., Coulembier O., Müller A.J. (2021). Accelerating the Crystallization Kinetics of Linear Polylactides by Adding Cyclic Poly (-Lactide): Nucleation, Plasticization and Topological Effects. Int. J. Biol. Macromol..

[28] Hsieh Y.-T., Nozaki S., Kido M., Kamitani K., Kojio K., Takahara A. (2020). Crystal Polymorphism of Polylactide and Its Composites by X-Ray Diffraction Study. Polym. J..

[29] Hoffman J.D., Weeks J.J. (1962). Melting Process and the Equilibrium Melting Temperature of Polychlorotrifluoroethylene. J. Res. Natl. Bur. Stan. Sect. A.

[30] Xiao H., Yang L., Ren X., Jiang T., Yeh J. (2010). Kinetics and Crystal Structure of Poly(Lactic Acid) Crystallized Nonisothermally: Effect of Plasticizer and Nucleating Agent. Polym. Compos..

[31] De Santis F., Pantani R., Titomanlio G. (2011). Nucleation and Crystallization Kinetics of Poly(Lactic Acid). Thermochim. Acta.

[32] Wu T.-M., Chen E.-C. (2006). Crystallization Behavior of Poly(ε-Caprolactone)/Multiwalled Carbon Nanotube Composites. J. Polym. Sci. Part. B: Polym. Phys..

[33] Guo X., Luo C., Fang M., Sun J., Chen M. (2023). Effects of Self-assembled Nucleating Agent on the Crystallization Behavior, Thermal Properties, and Mechanical Properties of Polylactic Acid. J. Appl. Polym. Sci..

[34] Sebek F.F.G., Nguon O.J., Bartos A., Brinke M.T., Van Drongelen M., Gojzewski H., Lefas J., Vancso G.J. (2024). Crystallization of Poly(Lactic Acid) Nucleated with the Sorbitol TBPMN. Polym. Test..

[35] Pantani R., De Santis F., Sorrentino A., De Maio F., Titomanlio G. (2010). Crystallization Kinetics of Virgin and Processed Poly(Lactic Acid). Polym. Degrad. Stab..

[36] Xu Y., Wang Y., Xu T., Zhang J., Liu C., Shen C. (2014). Crystallization Kinetics and Morphology of Partially Melted Poly(Lactic Acid). Polym. Test..

[37] D’Amico D.A., Cyras V.P., Manfredi L.B. (2014). Non-Isothermal Crystallization Kinetics from the Melt of Nanocomposites Based on Poly(3-Hydroxybutyrate) and Modified Clays. Thermochim. Acta.

